# Synesthesia and the McCollough Effect

**DOI:** 10.1177/2041669517711718

**Published:** 2017-06-01

**Authors:** V. S. Ramachandran, Zeve Marcus

**Affiliations:** Center for Brain and Cognition, University of California at San Diego, La Jolla, CA, USA

**Keywords:** color, imagery, memory, multisensory/cross-modal processing, neural mechanisms, perception, synesthesia

## Abstract

Synesthetes, who see printed black letters and numbers as being colored, are thought to have enhanced cross-activation between brain modules for color and form. Since the McCollough effect also results from oriented contours (i.e., form) evoking specific colors, we conjectured that synesthetes may experience an enhanced McCollough effect, and find that this is indeed true.

Synesthesia is a condition in which an otherwise normal person always sees printed letters or numbers tinged with specific colors (Baron-Cohen, Harrison, Goldstein, & Wyke, 1993; Cytowic, 1998; Galton, 1880; Ramachandran & Hubbard, 2001a, 2001b, 2003). This hereditary propensity (Asher et al., 2009; Barnett et al., 2008; Brang & Ramachandran, 2011) to make arbitrary links between sensations is thought to be caused by unusual cross-activation between brain regions that are specialized for different stimulus attributes and are ordinarily segregated from each other. For example: both V4, involved with processing color, and GA, or “grapheme area,” are in the fusiform gyrus and are connected more densely (axonally) in synesthetes (Rouw et al., 2007, 2011). The genes that produce such enhanced cross-activation between modules may also promote increased activation (Hubbard, Brang, & Ramachandran, 2011) or proliferation of pathways known to connect higher cortical visual areas to ones earlier in the hierarchy of visual processing (“back-projections”). These pathways have long been implicated in visual imagery—which involves sending visual information back from higher centers to Area 17 (Fellman & Van Essen, 1991; Klein et al., 2004) and which may explain why some synesthetes seem to have enhanced visual imagery (Brang & Ramachandran, 2010).

To explore these ideas further, we used the McCollough color aftereffect, or ME (McCollough, 1965), as a novel probe. Following adaptation to (say) a green and black pattern of vertical stripes, alternating every few seconds with a red and black pattern of horizontal stripes (Figure 1), an orientation-contingent color aftereffect is observed when black and white gratings are viewed. Vertical gratings are tinged with red and horizontal gratings with green. It is thought that prolonged exposure to green vertical stripes fatigues neurons sensitive to green vertical contours (Johnson, Hawken, & Shapley, 2008; McCollough, 1965), whereas viewing red horizontal stripes does the converse. Therefore, subsequent viewing of white vertical stripes causes a disproportionate activation of unadapted vertical-red neurons, causing you to see red (just as lukewarm water feels ice-cold if you have previously immersed your hand in hot water).

**Figure 1.**
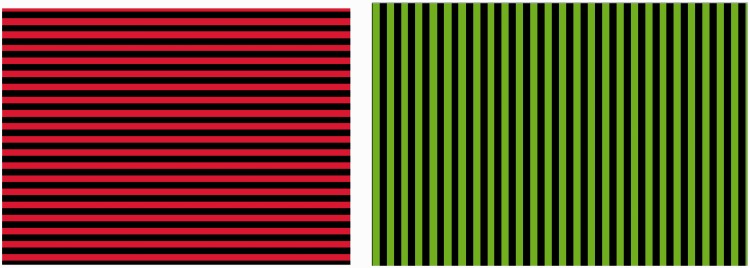
Induction images for the McCollough effect.

Since ME color is only seen when stripes are present and because there are no orientation selective cells in the retina—only in cortex—one could conclude that ME probably involves the selective adaptation of *double-duty* neurons in the cortex (Johnson et al., 2008; McCollough, 1965) sensitive to color and form.

During the last two decades, 30 or so topographically organized maps of visual space have been discovered in the primate brain, along with the numerous interconnections between them. Each map appears to be specialized for a specific attribute (e.g., color, motion, or depth). Although the areas are organized in a quasi-hierarchical manner, there are twice as many back-projection from any given area to areas earlier in the hierarchy (Felleman & Van Essen, 1991; Hinds et al., 2009; Shipp & Zeki 1989). Apart from speculations on their possible role in overall gain control, there have been few viable hypotheses of how these back-projections do their jobs (or, indeed, what their job is), although some steps forward have been taken in support of predictive coding models (Muckli et al., 2015).

Because ME and grapheme-color synesthesia both involve interactions between color and form (orientation), we wondered whether, in synesthetes, the vividness and persistence of the ME might be amplified due to the preexisting genetic propensity to link color and form in these individuals.

Seven individuals, who experienced synesthesia and displayed test–retest consistency, were chosen for the experiment (as were 13 nonsynethetes, recruited from an undergraduate pool). They were shown adaptation stimuli subtending 43°, consisting horizontal green [rgb(0, 255, 0), (120, 100%, 50%)]–black, and vertical red [rgb(255, 0, 0), hsl(0, 100%, 50%)]–black, square wave gratings (Figure 1), with a spatial frequency of .63 cycles per degree. The two frames alternated every 10 seconds for 3 minutes total.

After adaptation, subjects were shown a test display (Figure 2(a)), in which the top square does not produce an aftereffect on its own, because the subjects did not previously adapt to the 45° orientation. They used arrow keys to introduce color to the top square until it matched the strength of the ME below it (Figure 2(b)). They were tested every 5 minutes for 30 minutes. Effect percentages within subjects are calculated in relation to their first trial (their first match marks the initial strength of the effect—as in, 100% ME).

**Figure 2.**
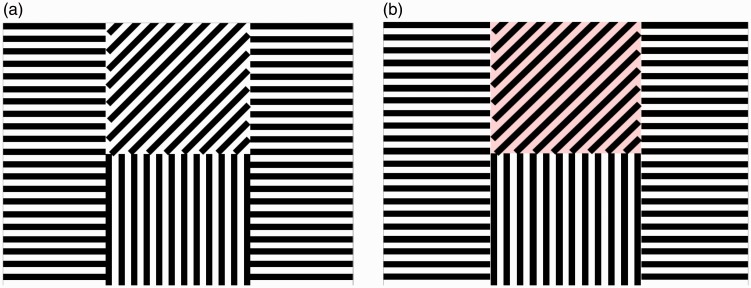
(a) Example of test pattern used to measure the McCollough effect. (b) Example of test pattern with colored-in matching stimulus.

Decay curves were recorded by plotting 100 × Me_t_/ME_0_ against t and compared with the normal decay curve given by Me_t_/ME_0_ = 1 − (1/12)t^1/3^ (Shute, 1979). Subjects in the synesthete group (*n* = 7) showed a striking (on average threefold) increase in persistence of the effect, when compared with nonsynesthete controls (*n* = 13, all having normal to corrected vision). Comparisons between the slopes of decay (d) for the groups showed a highly significant difference, *t*(17.9) = 4.57, *p* < .001; *p* = .00023, see Figure 3.

**Figure 3.**
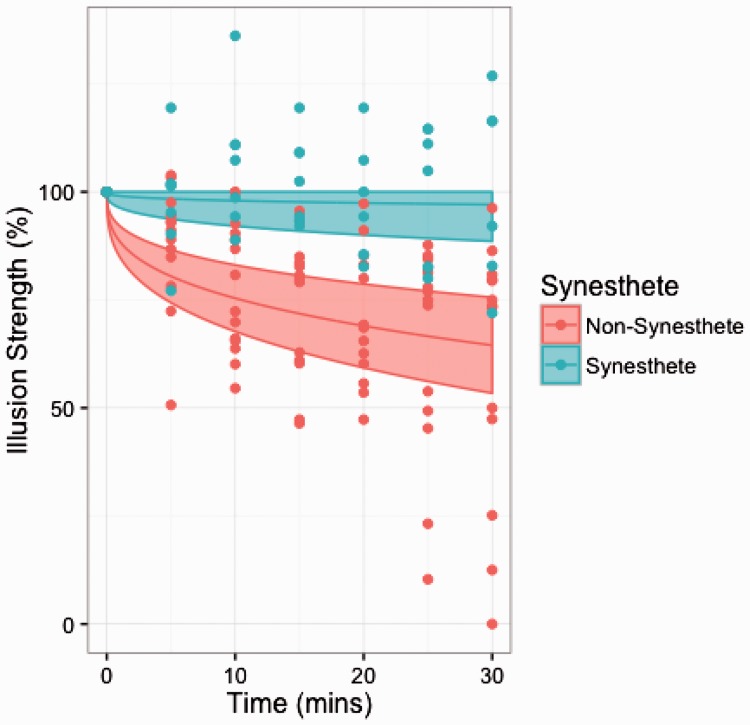
Visualized data for nonsynesthete (red) and synesthete (blue) ME decay over time (y). Groups' mean decay and 95% confidence intervals are represented linearly, individual's illusion strength at each test period are plotted with points.

For comparison, instead of ME, we used ordinary color aftereffects—which result from bleaching of cone pigments rather than adaptation of cortical neurons. We had 4 synesthetes and 13 nonsynesthete controls view a green heart, subtending 20°, for 90 seconds, and subsequently look at an outline of the heart on a homogenous blank screen. This procedure allowed us to measure the initial strength and duration of the effect using the same method of adjustment as before. There was an average of 29.5 seconds for the afterimage to decay fully in synesthetes and averaging 33 seconds with control subjects (Figure 4). Comparisons between the groups' afterimage durations (d) showed no significant difference, *t*(6.4153) = 0.46447, *p* > .001, *p* = .6577. That the aftereffect persisted for the same brief duration in both groups suggests that the increased persistence is specific to ME.

**Figure 4.**
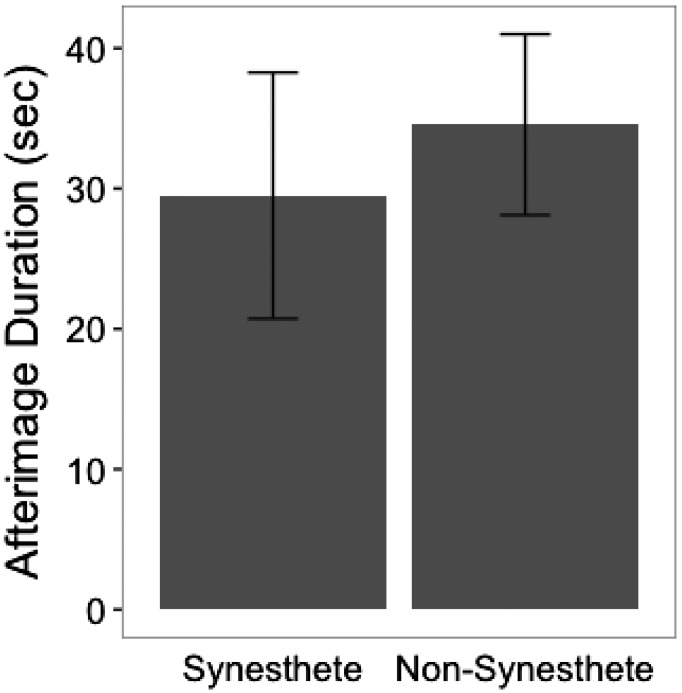
Synesthete and nonsynesthete afterimage durations (in seconds), with error bars.

Taken collectively, these observations are broadly consistent with the cross-activation theory of synesthesia. It is possible that the ME, which involves utilizing connectivity between form and color, can take advantage of the preexisting enhancement of such connections characterizing synesthetes. The net result is a more persistent ME. In both cases, the linking of color and form might be mediated either by lateral connections or by enhanced activity of back-projections. Such enhanced activity could set the stage for an echo-like reverberation of signals between areas—which is consistent with the known higher incidence of eidetic imagery in synesthesia (Brang & Ramachandran, 2010). These speculative conjectures can be tested using brain imaging (Brang, Hubbard, Coulson, Huang, & Ramachandran, 2010), which tend to have a hypnotic effect on scanners of the scientific literature.

Anomalous vision—whether studied in people with visual disturbances like synesthesia or in nonsynesthetes viewing visual illusions—can provide a valuable probe for exploring the cascade of activity in visual areas that culminates in our perception of the world.
